# Computational modelling of myocardial metabolism in patients with advanced heart failure

**DOI:** 10.1002/ejhf.3746

**Published:** 2025-07-15

**Authors:** Niklas Beyhoff, Vera M. Braun, Marieluise Kirchner, Lucy E.M. Finnigan, Christoph Knosalla, István Baczkó, Evgenij Potapov, Ulrich Kintscher, Tilman Grune, Titus Kuehne, Hermann‐Georg Holzhütter, Philipp Mertins, Damian J. Tyler, Hendrik Milting, Betty Raman, Oliver J. Rider, Stefan Neubauer, Nikolaus Berndt

**Affiliations:** ^1^ Division of Cardiovascular Medicine, Radcliffe Department of Medicine University of Oxford Oxford UK; ^2^ Department of Cardiology, Angiology and Intensive Care Medicine Deutsches Herzzentrum der Charité Berlin Germany; ^3^ Charité ‐ Universitätsmedizin Berlin, corporate member of Freie Universität Berlin and Humboldt‐Universität zu Berlin Berlin Germany; ^4^ Institute of Pharmacology, Max Rubner Center for Cardiovascular Metabolic Renal Research Charité ‐ Universitätsmedizin Berlin Berlin Germany; ^5^ DZHK (German Centre for Cardiovascular Research), Partner Site Berlin Berlin Germany; ^6^ Berlin Institute of Health at Charité ‐ Universitätsmedizin Berlin Berlin Germany; ^7^ Proteomics Platform Max Delbrück Center for Molecular Medicine (MDC) Berlin Germany; ^8^ Department of Cardiothoracic and Vascular Surgery Deutsches Herzzentrum der Charité Berlin Germany; ^9^ Department of Pharmacology and Pharmacotherapy, Albert Szent‐Györgyi Medical School University of Szeged Szeged Hungary; ^10^ German Institute of Human Nutrition Potsdam‐Rehbrücke Nuthetal Germany; ^11^ Institute of Computer‐assisted Cardiovascular Medicine Deutsches Herzzentrum der Charité Berlin Germany; ^12^ Institute of Biochemistry Charité ‐ Universitätsmedizin Berlin Berlin Germany; ^13^ Erich and Hanna Klessmann Institute for Cardiovascular Research and Development, Clinic for Thoracic and Cardiovascular Surgery Heart and Diabetes Center NRW Bad Oeynhausen Germany

**Keywords:** Cardiomyopathy, Computational modelling, Heart failure, Metabolism, Precision medicine, Proteomics, Ventricular assist device

## Abstract

**Aims:**

Perturbations of myocardial metabolism and energy depletion are well‐established hallmarks of heart failure (HF), yet methods for their systematic assessment remain limited in humans. This study aimed to determine the ability of computational modelling of patient‐specific myocardial metabolism to assess individual bioenergetic phenotypes and their clinical implications in HF.

**Methods and results:**

Based on proteomics‐derived enzyme quantities in 136 cardiac biopsies, personalised computational models of myocardial metabolism were generated in two independent cohorts of advanced HF patients together with sex‐ and body mass index‐matched non‐failing controls. The bioenergetic impact of dynamic changes in substrate availability and myocardial workload were simulated, and the models' ability to predict the myocardial response following left ventricular assist device (LVAD) implantation was assessed. Compared to controls, HF patients had a reduced ATP production capacity (*p* < 0.01), although there was remarkable interindividual variance. Utilisation of glucose relative to fatty acids was generally higher in HF patients, depending on substrate availability and myocardial workload. The ratio of fatty acid to glucose utilisation was associated with reverse cardiac remodelling after LVAD implantation and highly predictive of an improvement in left ventricular ejection fraction ≥10% (C‐index 0.94 [0.81–1.00], *p* < 0.01). System‐level simulations identified fatty acid administration and carnitine supplementation in those with low mitochondrial carnitine content as potential pharmacological interventions to restore myocardial substrate utilisation.

**Conclusions:**

Computational modelling identified a subset of advanced HF patients with preserved myocardial metabolism despite a similar degree of systolic dysfunction. Substrate preference was associated with the myocardial response after LVAD implantation, which suggests a role for substrate manipulation as a therapeutic approach. Computational assessment of myocardial metabolism in HF may improve understanding of disease heterogeneity, individual risk stratification, and guidance of personalised clinical decision‐making in the future.

## Introduction

The energetic demands of the human heart are met by a complex network of interrelated metabolic pathways that enable utilisation of a variety of energy‐delivering substrates.^1^, ^2^, ^3^, ^4^ The large amounts of ATP required to maintain cardiac function are predominantly produced via oxidation of fatty acids (FAs) and glucose (Glc), but the heart can also process ketone bodies, amino acids, and lactate in response to changes in workload, substrate availability, or hormonal status.^1^ This is achieved through a tightly regulated system of enzymes involved in the uptake, breakdown, and utilisation of substrates as well as the generation and transfer of energy.^1^, ^2^, ^3^ While derangements of myocardial metabolism and energy depletion are well‐established hallmarks of heart failure (HF),^1^, ^2^, ^3^, ^4^ methods for the systematic assessment of the complex and dynamic metabolic network in humans remain limited.

Computational modelling of organ functions in individual patients (‘digital twins’) represents an emerging precision medicine approach for personalised diagnosis of specific disease mechanisms and tailored clinical decision‐making.^5^, ^6^ We recently developed a comprehensive mathematical model of myocardial metabolism that recapitulates experimentally observed physiology in cultured cardiomyocytes, isolated perfused animal hearts, and *in vivo* studies in humans.^7^ Based on proteomics‐derived enzyme quantity profiles from myocardial biopsies, the model can reconstruct all main pathways through which energy‐delivering substrates are processed to generate ATP.^7^ This approach allows quantification of ATP production capacity at the single patient level and also facilitates simulations of the individual bioenergetic response to dynamic changes in substrate availability, myocardial workload, and enzyme activity.^7^

In the present study, this computational framework was applied to reconstruct patient‐specific cardiac bioenergetic phenotypes in HF. By comparing two independent cohorts of non‐failing controls and patients with advanced HF of different aetiology and various cardiometabolic comorbidities, we aimed to define the broad spectrum of metabolic alterations in the failing heart and to evaluate this novel approach with regard to personalised outcome predictions and customised target discovery.

## Methods

The study was approved by the Ethics Committee of Charité ‐ Universitätsmedizin Berlin, Germany (EA4/124/21) and was conducted in compliance with the Declaration of Helsinki.

### Myocardial tissue acquisition

Human left ventricular (LV) biopsies were procured for research purposes under approval from local ethics committees (EA4/028/12 and EA4/124/21, Charité ‐ Universitätsmedizin Berlin, Germany; 21/2013, Ruhr‐University Bochum, located in Bad Oeynhausen, Germany; 4991‐0/2010‐1018EKU [339/PI/010], and Scientific and Research Ethical Committee of the Medical Scientific Board at the Hungarian Ministry of Health [ETT‐TUKEB], Hungary). Non‐failing myocardium was obtained from organ donors with no previous history of cardiovascular disease whose hearts could not have been used for transplantation due to non‐medical reasons. Surgical biopsies from failing myocardium were collected from the apical core during implantation of a left ventricular assist device (LVAD) or from explanted hearts during organ transplantation. Samples from non‐failing organ donors were procured under national presumed consent legislation in accordance with ETT‐TUKEB regulations; all participants in the HF group provided written informed consent. Samples were stored in liquid nitrogen or at −80°C until further processing.

### Quantitative proteomics of myocardial tissue

Label‐free global protein quantification was performed by liquid chromatography with tandem mass spectrometry analyses as described before.^7^, ^8^ Corresponding sample preparation, data acquisition, and statistics are detailed in online supplementary *Appendix* *S1*. Only samples containing <5% haemoglobin and <5% collagen fraction were included to control for potential blood contamination and presence of excessive scar tissue, respectively.

### Computational modelling of myocardial metabolism

We previously developed and validated a kinetic model of myocardial metabolism (CARDIOKIN1) encompassing 296 metabolic processes (biochemical reactions) involved in 172 distinct metabolic functions.^7^ Detailed information on the computational methods can be found in online supplementary *Appendix* *S1* and our previous work introducing the model.^7^ Briefly, the model simulates the catabolism of FAs, Glc, lactate/pyruvate, ketone bodies, and branched‐chain amino acids for energy production. It incorporates transmembrane ion transport, mitochondrial electrophysiology, and short‐term regulatory effects of insulin and catecholamines.^7^ The model's dynamics are described by first‐order differential equations, with ion fluxes modelled using Goldman–Hodgkin–Katz equations.^9^ Enzyme and transporter rate laws were derived from literature or experimental data, accounting for substrate/product regulation, allosteric effects, and phosphorylation as previously shown.^10^

Personalised models were constructed by integrating individual proteomics‐derived protein intensities from LV biopsies.^7^, ^11^ Enzyme/transporter activities (*V*_max_) were scaled using individual enzyme abundances:

$$V_{\max}^{\text{subject}}=V_{\max}^{\text{normal}}\;\frac{E^{\text{subject}}}{E^{\text{control}}}$$

where *E* ^control^ represents the mean enzyme intensity in controls, and *E* ^subject^, the individual's enzyme intensity.^7^, ^11^ Missing values were imputed using group averages or assumed unchanged from controls.^7^ Reference *V*_max_ values were derived by fitting the model to experimental data for non‐failing hearts.^7^

The individual metabolic response to an increased ATP demand was evaluated by simulating the temporal changes in metabolic state induced by a rise in ATP consumption above resting levels. The ATP consumption rate was modelled using a generic hyperbolic rate law:

$$v_{\mathrm{ATP}}=k_{\text{load}}\cdot \frac{\mathrm{ATP}}{\mathrm{ATP}+K_{m}}.$$

The parameter $k_{\text{load}}$ was stepwise increased until the ATP production rate converged to its maximal value.

All computations were performed using MATLAB (release R2023b; MathWorks, Natick, MA, USA).

### Clinical data and endpoint definitions

Demographics, clinical information, and outcome data were collected from patients' medical records. Obesity was defined as body mass index (BMI) ≥30 kg/m^2^ according to the World Health Organization. Echocardiography was performed as part of clinical routine care. Clinical outcomes of interest were the myocardial structural and functional response after LVAD implantation, defined as the echocardiographic change in LV end‐diastolic diameter (LVEDd) and LV ejection fraction (LVEF) between preoperative examination and after LVAD implantation.^12^, ^13^, ^14^ Reverse cardiac remodelling was classified according to the Utah‐Inova stages.^12^ Optimal mechanical unloading was defined as a relative reduction in LVEDd ≥15% from pre‐ to post‐LVAD.^13^, ^14^

### Confirmation cohort

For external confirmation, publicly available data were leveraged from a comprehensive multi‐omics study conducted by the Sydney heart bank (accessible via the PRIDE/ProteomeXchange repository with project identifier PXD018678).^15^ Detailed methodology is outlined in online supplementary *Appendix* *S1*.

### Statistics

Data are presented as mean ± standard deviation or median [95% confidence interval]. Normality was tested using the Kolmogorov–Smirnov test. Two‐tailed unpaired Student's *t*‐test or Mann–Whitney U test was used to compare two groups, depending on data distribution, while categorical variables were compared with the chi‐squared test. One‐way ANOVA with Tukey's post‐hoc test was applied for multiple group comparisons. Linear regression (with 95% confidence interval) and Pearson's correlation coefficient assessed relationships between continuous variables. The relationship between clinical/demographic features and metrics of myocardial metabolism was analysed by multiple linear regression (reporting standardised beta coefficients as absolute numbers for easy comparisons between the independent variables regardless of different units of measurement and positive/negative values, respectively). Receiver‐operating characteristic curve analysis determined discriminatory performance for structural/functional response after LVAD placement. A *p*‐value of < 0.05 was considered statistically significant. Analyses were performed using GraphPad PRISM 9 and IBM SPSS (version 29.0.1.0).

## Results

### Study populations, tissue acquisition, and model generation

**Figure 1:**
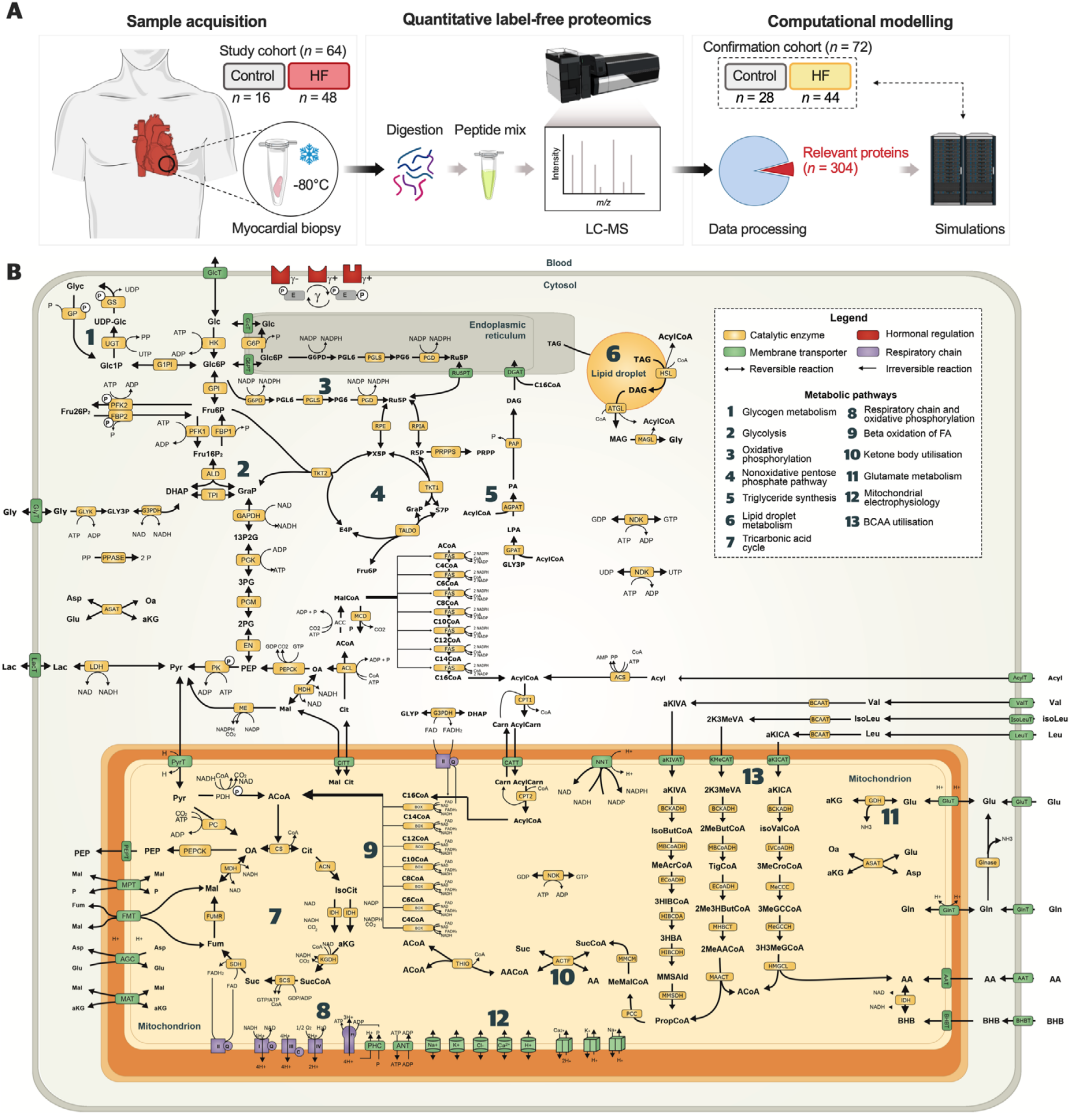
Study workflow and reaction scheme of the computational model. (*A*) Myocardial biopsies were analysed by liquid chromatography‐mass spectrometry (LC‐MS) for untargeted quantitative label‐free proteomics. Individual intensity profiles of relevant metabolic proteins were used to reconstruct patient‐specific myocardial metabolism via computational modelling. Results were validated in an independent proteomics dataset. (*B*) Reaction scheme of the computational model. The full abbreviation list, kinetic rate laws of reaction rates, and derivation of metabolite concentrations were previously described.^7^ Modified from Berndt *et al*.^7^

Patient‐specific computational models of myocardial metabolism were generated based on a total of 136 LV biopsies leveraging data from two different trials conducted in Europe (study cohort; *n* = 64) and Australia (confirmation cohort; *n* = 72).^15^ Design and workflow of the study are illustrated in *Figure* *1A*.

Demographics and clinical characteristics of the study cohort are summarised in *Table* *1*. Forty‐eight patients with advanced HF (LVEF 20 ± 10%; 52.1% female) were compared to 16 non‐failing sex‐ and BMI‐matched controls. HF patients were older (*p* < 0.05) and had a high prevalence of cardiometabolic comorbidities (*Table* *1*). Causes of HF included ischaemic (27.1%) and non‐ischaemic cardiomyopathy (72.9%). The majority was New York Heart Association (NYHA) functional class IV despite guideline‐directed medical therapy; 48.9% had atrial fibrillation.

**Table 1:** Demographic and clinical characteristics of the study cohort

	Control	HF	*p*‐value*
*n*	16	48	
Sample acquisition			
Explant, %	100 (16/16)	52.1 (25/48)	
LVAD implantation, %	0 (0/16)	47.9 (23/48)	
Demographics			
Age, years	44 ± 15	54 ± 26	0.025
Female sex, %	43.8 (7/16)	52.1 (25/48)	0.77
BMI, kg/m^2^	24.7 ± 4.8	26.0 ± 4.7	0.37
Obesity, %	14.3 (2/14)	17.0 (8/47)	0.81
Diabetes mellitus, %	0 (0/16)	29.8 (14/47)	
Arterial hypertension, %	31.3 (5/16)	36.2 (17/47)	0.72
Dyslipidaemia, %	–	53.2 (25/47)	
Atrial fibrillation, %	–	48.9 (22/45)	
HF aetiology			
Ischaemic cardiomyopathy, %	0 (0/16)	27.1 (13/48)	
Non‐ischaemic cardiomyopathy, %	0 (0/16)	72.9 (35/48)	
Clinical characteristics pre‐surgery			
NYHA class III, %	–	38.1 (16/42)	
NYHA class IV, %	–	61.9 (26/42)	
LVEF, %	–	20 ± 9	
Systolic blood pressure, mmHg	122 ± 21	106 ± 15	0.003
Diastolic blood pressure, mmHg	78 ± 18	68 ± 13	0.023
Heart rate, bpm	–	85 ± 16	
Medication			
Beta‐blocker, %	0 (0/16)	91.1 (41/45)	
ACEi or ARB, %	18.8 (3/16)	80.0 (36/44)	
MRA, %	0 (0/16)	76.2 (32/42)	
SGLT2i, %	0 (0/16)	2.2 (1/45)	
Diuretic therapy, %	0 (0/16)	95.6 (43/45)	
Statin, %	0 (0/16)	31.1 (14/45)	
Insulin, %	0 (0/16)	13.3 (6/45)	

ACEi, angiotensin‐converting enzyme inhibitor; ARB, angiotensin receptor blocker; BMI, body mass index; HF, heart failure; LVAD, left ventricular assist device; LVEF, left ventricular ejection fraction; MRA, mineralocorticoid receptor antagonist; NYHA, New York Heart Association; SGLT2i, sodium–glucose co‐transporter 2 inhibitor.

* Statistical significance was determined using unpaired Student's *t*‐test, Mann–Whitney U test, or chi‐squared test.

Left ventricular biopsies were obtained during LVAD implantation (47.9% of HF patients) or from explanted hearts (remaining subjects). Individual myocardial protein intensity profiles were assessed by tandem mass spectrometry‐based quantitative proteomics, providing a deep heart proteome dataset with 8419 distinct protein groups identified, and a uniform coverage across all samples with an average of 5079 ± 123 proteins quantified per sample (online supplementary *Figure* *S1*). A total of 4304 protein groups had more than 70% valid values over all samples and were used for further analyses. Out of these 4304 entries, quantification intensities of 304 proteins involved in myocardial metabolism (expression heatmap displayed in online supplementary *Figure* *S2*) were used for customised model generation. The final models comprised 296 different biochemical reactions in four (sub‐)cellular compartments that were successfully reconstructed in >95% of the study cohort (*Figure* *1B*).

### Characterisation of myocardial metabolism in advanced heart failure

**Figure 2:**
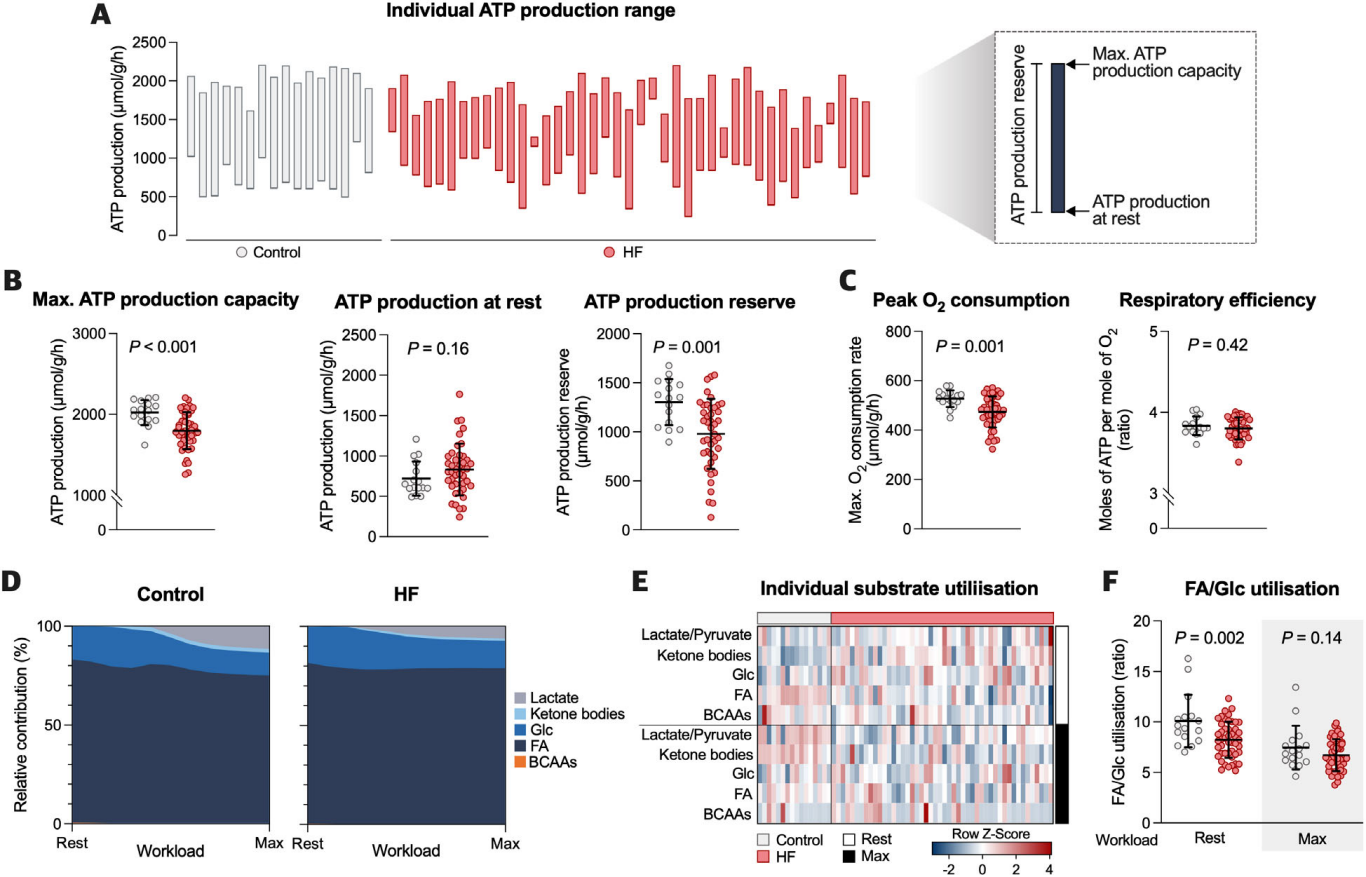
Characterisation of myocardial metabolism during fasting state. (*A*) Diagram showing individual energy consumption rates. The top values of the bars indicate maximal ATP production capacity, bottom values refer to ATP production at rest. The bar length corresponds to ATP production reserve (the difference between maximal ATP production capacity and resting ATP production). (*B*) Maximal ATP production capacity, resting ATP production, and ATP production reserve per group. (*C*) Peak oxygen consumption rate and amount of ATP produced per mole of oxygen. (*D*) Relative contribution of the energy‐delivering substrates to total energy expenditure at different levels of myocardial workload (expressed as group means of ATP equivalents). (*E*) Substrate flux rates during rest and maximal workload. (*F*) Ratio of fatty acid (FA) to glucose (Glc) utilisation at rest and during maximal workload per study group. Statistical significance was determined using a two‐tailed Student's *t*‐test (*B*, *C*, *F*). BCAA, branched‐chain amino acid; HF, heart failure.

First, we mapped the myocardial metabolic network considering physiological substrate availability following overnight fasting (*Figure* *2*). Maximal ATP production capacity and reserve were diminished in patients with HF, whereas ATP production at rest was similar compared to controls (*Figure* *2A,B*). The reduced maximal ATP production capacity was associated with a lower peak oxygen consumption rate in HF (*p* = 0.001), although respiratory efficiency, that is, the amount of oxygen used to generate one mole of ATP, did not differ significantly (*Figure* *2C*). Based on metabolic flux rate analyses, we estimated the relative contribution of the different substrates to ATP production at varying myocardial workload (*Figure* *2D,E*). Interindividual substrate utilisation was highly heterogeneous (*Figure* *2E*). Oxidation of FAs represented the main source of energy at all stages of myocardial workload in both non‐failing and failing hearts (*Figure* *2D*). The contribution of ketone bodies and lactate/pyruvate to resting energy production was only marginal but increased with higher myocardial workload (*Figure* *2D*). Utilisation of amino acids accounted for <1% of resting ATP synthesis and decreased even further at higher workloads (*Figure* *2D*).

As a simplified estimate of myocardial substrate preference, we determined the ratio of FA to Glc utilisation (*Figure* *2F*). HF patients showed a diminished use of FAs with a shift towards Glc oxidation at rest (*Figure* *2E,F*). No group differences were observed in FA/Glc utilisation during maximal myocardial workload (*Figure* *2F*).

### Interindividual variability in myocardial energetic state

**Figure 3:**
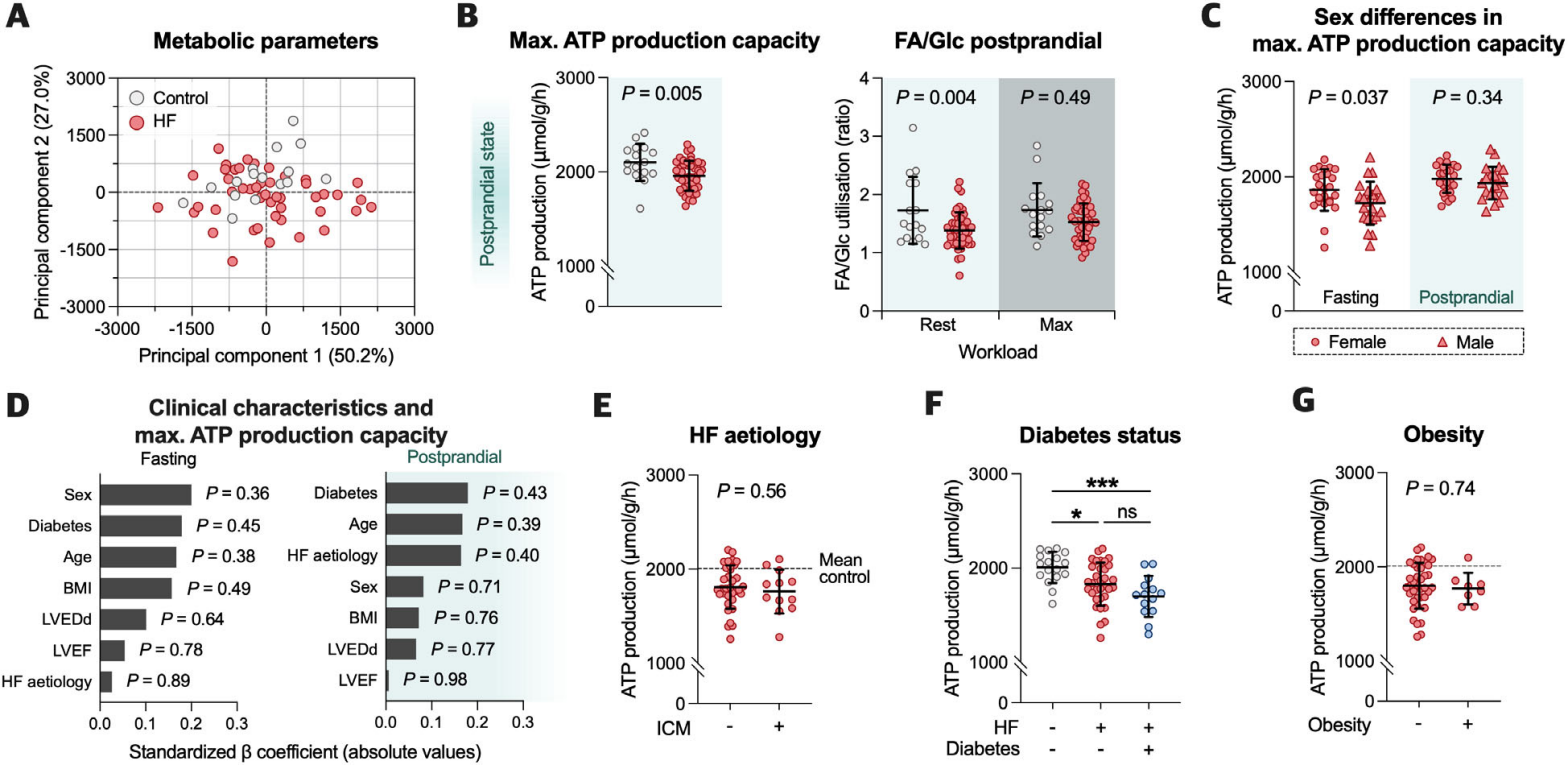
Metabolic landscape in heart failure (HF). (*A*) Principal component analysis of key metabolic parameters (*n* = 118 different metabolic readouts). (*B*) Maximal ATP production capacity (left) and the ratio of fatty acid (FA) to glucose (Glc) utilisation (right) at physiologic postprandial substrate composition. (*C*) Comparison of maximal ATP production capacity between female and male patients with HF (in the physiologic fasting and postprandial state, respectively). (*D*) Standardised beta coefficients from multiple linear regression models describing clinical characteristics and maximal ATP production capacity. None of the clinical characteristics' coefficients reached statistical significance indicating no relevant association with maximal ATP production capacity. (*E*) Analysis of maximal ATP production in patients with HF according to underlying HF aetiology (ischaemic cardiomyopathy [ICM] vs. non‐ICM). (*F*) Computation of maximal ATP production capacity considering reported differences in circulating substrate/hormone/ion concentrations after overnight fasting between diabetic and non‐diabetic individuals. For non‐failing controls and HF patients without a history of diabetes, physiologic concentrations were simulated. Computations for HF patients with diabetes were performed assuming fasting concentrations reported in diabetic individuals. (*G*) Maximal ATP production capacity in HF patients stratified according to body mass index (BMI) (obese vs. non‐obese). Statistical significance was determined using a two‐tailed Student's *t*‐test (*B*, *C*, *E*, *F, G*), multiple linear regression (*D*), and one‐way ANOVA with multiple comparisons (*F*). LVEDd, left ventricular end‐diastolic diameter; LVEF, left ventricular ejection fraction.

Despite similar degrees of systolic dysfunction, principal component analysis of 118 key metabolic parameters (detailed in online supplementary *Table* *S1*) revealed considerable interindividual variability in myocardial energetics among HF patients, with >75% of variance explained by the first two principal components but no clear group clustering (*Figure* *3A*). To gain insights into the influence of circulating nutrient levels on bioenergetics, the individual metabolic response to changes in substrate availability was simulated (*Figure* *3B*). Maximal ATP production capacity and FA/Glc utilisation at rest were consistently lower in HF patients than in controls when exposed to either physiological postabsorptive (*Figure* *2B,E,F*) or postprandial fuel compositions (*Figure* *3B*). Simulations were also performed considering reported differences in fasting substrate/hormone/ion concentrations between control subjects and HF patients,^16^ but revealed no major implications for overall ATP production or respiratory efficacy (online supplementary *Figure* *S3*).

At fasting state, maximal ATP production capacity was slightly lower in males compared to female HF patients (*Figure* *3C*). However, multiple linear regression analyses indicated no significant effects of demographic or clinical characteristics on energy production capacity (*Figure* *3D*), which was also independent of HF aetiology (*Figure* *3E*). Given the profound metabolic impact of diabetes mellitus, the models were further adjusted to consider differences in the fasting plasma profile of diabetic and non‐diabetic individuals (*Figure* *3F*). HF patients with diabetes tended to have an even lower ATP production capacity but differences failed to reach statistical significance (*Figure* *3F*). Obesity had no major impact on maximal ATP production capacity in this cohort of advanced HF patients (*Figure* *3G*).

### External confirmation of metabolic phenotypes in heart failure

For external confirmation of findings, an independent set of computational models was created based on myocardial proteomics data from an Australian cohort of patients with advanced HF (*n* = 44 samples) and non‐failing controls with similar age, sex, and BMI (*n* = 28)^15^ (*Figure* *1A*, online supplementary *Figure* *S4A*). Ischaemic and (non‐ischaemic) dilated cardiomyopathy each accounted for approximately half of HF cases in the confirmation cohort, all of whom were NYHA functional class III/IV and had severely reduced systolic function.^15^

Overall, there was good agreement between both cohorts regarding effect direction and size, confirming the robustness of the main findings (online supplementary *Figure* *S4B–D*). In line with the study cohort, metabolic state was highly heterogeneous among HF patients in the confirmation cohort (online supplementary *Figure* *S4B*). Maximal ATP production capacity was consistently reduced across HF populations (online supplementary *Figure* *S4C*). Furthermore, features of oxygen consumption, respiratory efficiency, and myocardial substrate preference were replicated in the confirmation cohort (online supplementary *Figure* *S4C*). This high consistency suggested a general validity of these findings in patients with advanced HF and underscored the good reproducibility of the computational approach.

### Myocardial substrate preference predicts ventricular response after left ventricular assist device implantation

**Figure 4:**
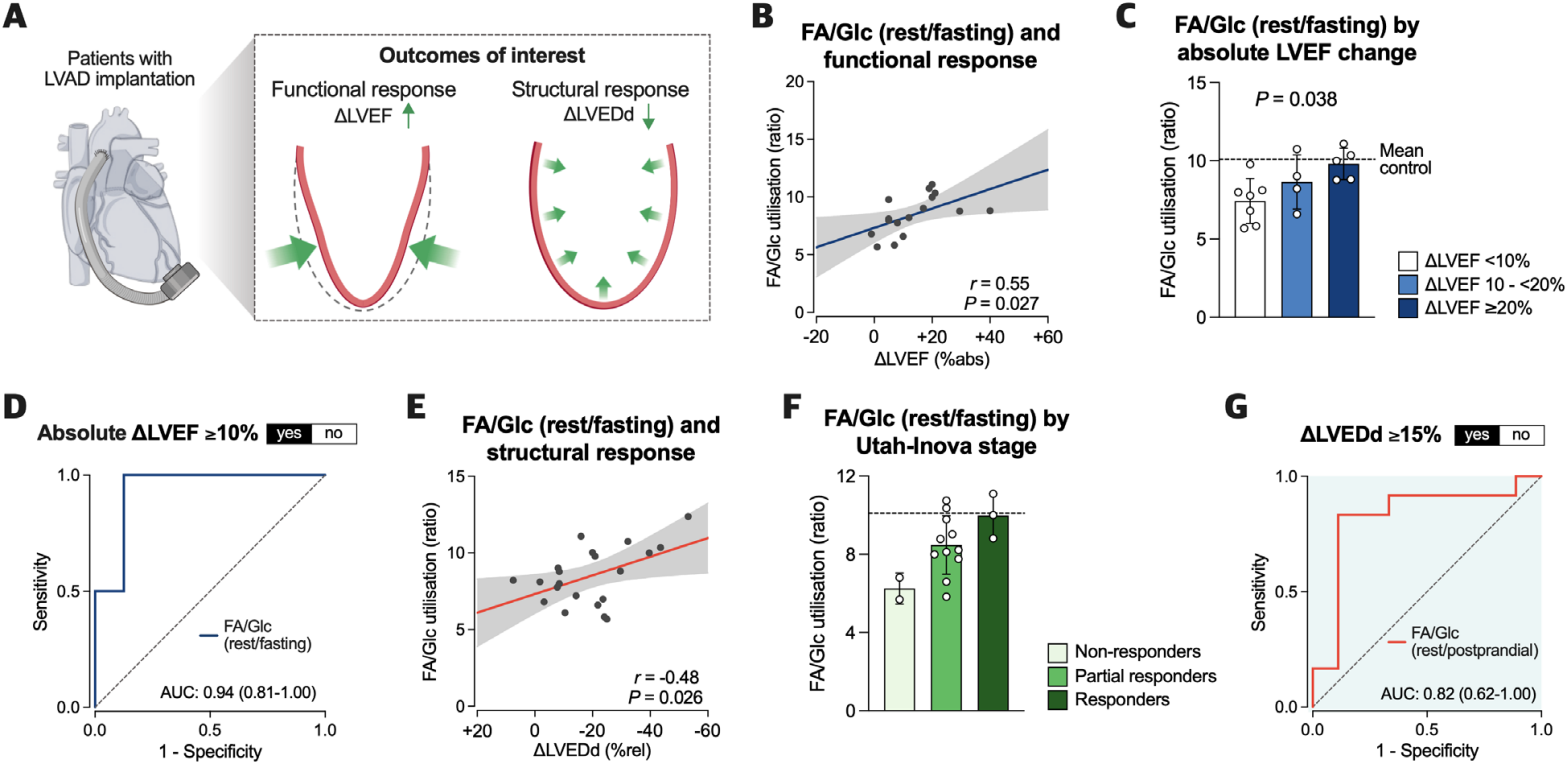
Association between myocardial substrate preference and outcome after left ventricular assist device (LVAD) implantation. (*A*) Illustration of myocardial functional and structural response after LVAD implantation as clinical outcomes of interest. (*B*) Correlation between resting fatty acid to glucose (FA/Glc) ratio at fasting state and the absolute change in left ventricular ejection fraction (LVEF) after LVAD implantation. (*C*) FA/Glc utilisation at rest/fasting stratified by absolute change in LVEF. (*D*) Receiver‐operating characteristic curve of FA/Glc utilisation at rest/fasting to predict an absolute improvement in LVEF ≥10% following LVAD. (*E*) Correlation between FA/Glc utilisation at rest/fasting and the relative change in left ventricular end‐diastolic diameter (LVEDd) from pre‐ to post‐LVAD. (*F*) FA/Glc utilisation at rest/fasting according to Utah‐Inova stage. (*G*) Receiver‐operating characteristic curve of resting FA/Glc at postprandial state to distinguish patients with a relative reduction in LVEDd ≥15%. *n* = 16–22. Statistical significance was determined using Pearson correlations (*B*, *E*), one‐way ANOVA (*C*), and receiver‐operating characteristics (*D*, *G*). AUC, area under the curve.

To assess the impact of bioenergetic phenotypes on clinical outcomes, we tested whether metabolic parameters were associated with the myocardial functional and/or structural response following LVAD implantation (*Figure* *4A*). Data on the change in LVEF (functional response) and LVEDd (structural response) from pre‐ to post‐LVAD was available in 16/22 (72.7%) and 21/22 patients (95.5%), respectively. Median time between surgery and follow‐up echocardiography was 42 [9–141] weeks.

There was a linear relationship between resting FA/Glc ratio at fasting state and the absolute change in LVEF after LVAD implantation (*Figure* *4B*). When stratified by LVEF increase, patients with greater functional response showed significantly higher FA/Glc utilisation than those with only minor or no improvement (*Figure* *4C*). Based on receiver‐operating characteristic analysis, the FA/Glc ratio at rest/fasting yielded excellent accuracy to predict an absolute increase in LVEF ≥10% after LVAD implantation (*Figure* *4D*). Similarly, preserved substrate use was associated with the structural response upon LVAD support, where higher FA/Glc ratios were linked to more pronounced decreases in LVEDd (*Figure* *4E*). The same pattern was observed when reverse cardiac remodelling was classified according to the Utah‐Inova stages^12^ (*Figure* *4F*). FA/Glc utilisation at rest/fasting gradually increased from non‐responders and partial responders to nearly normal values in patients classified as responders (*Figure* *4F*). Furthermore, resting FA/Glc utilisation at post‐prandial state demonstrated a high accuracy to predict optimal mechanical unloading, defined as a relative reduction in LVEDd ≥15% (*Figure* *4G*).

### Personalised target discovery to manipulate myocardial substrate use

**Figure 5:**
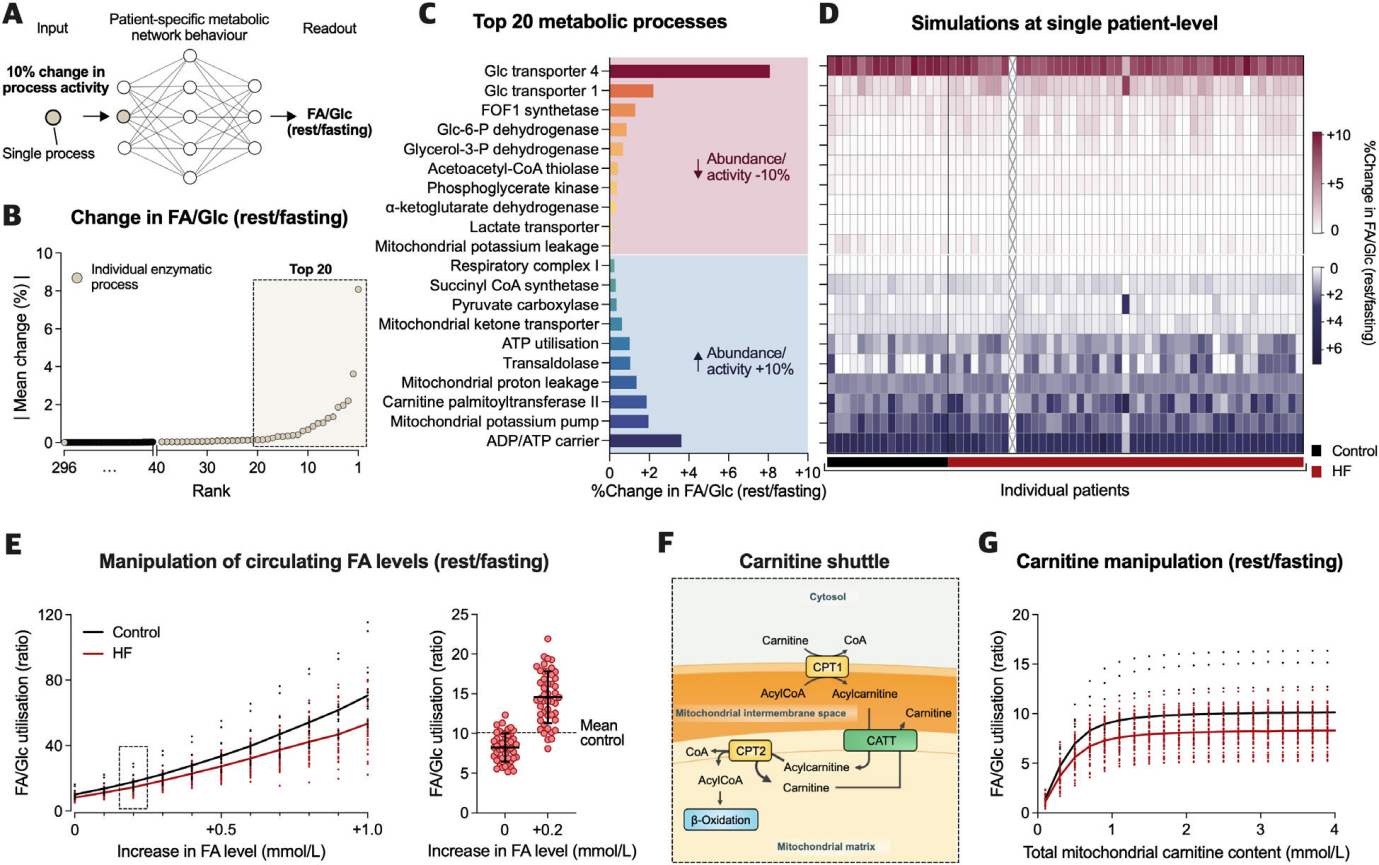
Patient‐specific target discovery to increase myocardial fatty acid/glucose (FA/Glc) utilisation. (*A*) Illustration of the applied metabolic control theory to explore molecular targets by simulating the impact of a 10% change in each individual metabolic process (*n* = 296) on the resting FA/Glc ratio. All simulations were performed considering substrate concentrations during physiological fasting state. (*B*) Metabolic functions/proteins ranked by absolute mean control coefficient. (*C*) Absolute mean control coefficient of the top 10 candidates yielding the highest absolute mean control coefficient by either reducing (upper panel) or enhancing their activity/abundance by 10% (lower panel). (*D*) Heat map depicting control coefficients of the candidates in *C* at the single patient level. (*E*) Relationship between increases in circulating FA levels and FA/Glc ratios (left graph), and comparison of predicted FA/Glc utilisation in heart failure (HF) patients at fasting state (0.5 mmol/L) and after increasing FA availability by 0.2 mmol/L (right graph). (*F*) Illustration of carnitine shuttle. (*G*) Relationship between total mitochondrial carnitine content and FA/Glc ratio. A physiological carnitine content of 3.0 mmol/L was considered as default for the computational model. CATT, carnitine O‐acetyltransferase; CoA, coenzyme A; CPT, carnitine palmitoyltransferase.

Given the observed association between preserved FA/Glc utilisation and favourable outcomes in LVAD patients, potential therapeutic strategies were identified to restore myocardial substrate preference (*Figure* *5*). While higher FA/Glc ratios could result from either enhancing FA use (increasing the numerator) or reducing Glc use (decreasing the denominator), the efficacy of manipulating underlying metabolic pathways may differ across both protein candidates and individual patients. Therefore, the effect of a 10% change in the activity of 296 distinct metabolic processes on the resting FA/Glc ratio at the single patient level was systematically explored (*Figure* *5A–D*). The highest predicted increase in FA/Glc use was achieved by either inhibiting insulin‐mediated Glc uptake via GLUT4 transporters or by enhancing mitochondrial ADP/ATP carrier (adenine nucleotide translocator) activity leading to higher cytosolic ATP concentrations, which in turn inhibit Glc utilisation (*Figure* *5C,D*).

Next, stepwise increases in circulating FA levels were simulated, which were associated with markedly higher FA/Glc ratios (*Figure* *5E*). Raising plasma FA concentration from 0.5 mmol/L during fasting state to 0.7 mmol/L corrected FA/Glc ratios to mean normal values in most HF patients (*Figure* *5E*). Interestingly, differences in substrate utilisation between controls and HF became even more pronounced at greater FA availability (*Figure* *5E*).

In addition, carnitine was explored which plays a critical role in FA use by enabling the transport of Acyl‐CoA from the cytosol into mitochondria (*Figure* *5F*). Simulations of varying mitochondrial carnitine content showed that carnitine supplementation above a certain saturation level may not result in further increases in FA/Glc ratios (*Figure* *5G*). Conversely, carnitine deficiency was predicted to have a more severe impact on FA utilisation in HF patients than controls, highlighting a potential role of supplementation therapy in those with diminished myocardial carnitine content (*Figure* *5G*).

## Discussion

This study combined two of the largest series of cardiac proteomics data reported in human HF to demonstrate the translational utility of patient‐specific computational modelling of myocardial metabolism. Derived from integration of personalised metabolic phenotyping, large‐scale computer simulations, and clinical information, three key conclusions were drawn (*Graphical Abstract*). First, myocardial bioenergetics are heterogeneous in advanced HF patients, ranging from severe derangements of the entire metabolic network to largely normal physiological states. Reduced ATP production capacity and a switch in myocardial substrate preference from FA to Glc utilisation represent general features of HF that can be quantitatively assessed in patients using computational modelling. Second, a potential link between myocardial substrate preference and the adaptive response after LVAD implantation has been identified, where patients with more normal FA/Glc utilisation showed favourable outcomes following mechanical circulatory support. This suggests that fuel choice plays a critical role in myocardial reverse remodelling, which may hold clinical significance as a therapeutic target in the future. Lastly, system‐level simulations of myocardial metabolism may enable personalised target discovery and prediction of individual treatment responses, e.g. to manipulate substrate use in the failing heart. As such, increasing FA availability and carnitine supplementation were identified as potential strategies to correct abnormal substrate preference in HF.

More than 80 years ago, Herrmann and Decherd proposed the concept of the failing heart as “an energy‐starved engine out of fuel”.^17^ Since then, multiple studies have confirmed deranged bioenergetics as a fundamental characteristic of HF pathophysiology, mainly based on altered expression of key metabolic enzymes and lower levels of energy‐rich phosphates in the failing myocardium.^1^, ^2^, ^3^, ^4^, ^15^, ^18^, ^19^ More recently, comprehensive interrogations via high‐throughput omics technologies became available enabling large‐scale quantification of transcripts, proteins, and metabolites.^15^, ^19^ While these methods yield detailed insights into molecular signatures, the static output produced lacks information about the dynamic network behaviour of metabolic systems, which is required to infer complex readouts like ATP production capacity. By integrating large biological datasets and kinetic information, mathematical models represent transformative precision medicine tools to gain a functional, quantitative, and dynamic network view on patient‐specific organ pathologies.^7^, ^11^ Our *in silico* findings highlight a remarkable interindividual variability in cardiac metabolic phenotypes in HF, even in patients with a similar degree of end‐stage disease. It appears likely that this high heterogeneity provides an explanation for the broad range of (partly conflicting) observations reported in HF patients and experimental models. Importantly, it also indicates a potential role for personalised approaches to inform HF diagnosis and treatment based on the individual metabolic phenotype.

The existing evidence on metabolic changes in the failing heart is somewhat controversial, especially with regard to myocardial substrate utilisation and corresponding metabolic interventions.^3^, ^19^, ^20^, ^21^, ^22^ In line with previous research,^1^, ^2^, ^18^ HF patients showed a switch from FA towards Glc use in the present study. By applying computational modelling, multiple biological determinants of myocardial substrate use were considered, including the individual protein profile, circulating metabolite concentrations, different myocardial workloads, and biochemical/biophysical interactions, thereby supporting experimental findings obtained with conventional methods.^18^ Myocardial ketone metabolism recently emerged as a potential target for therapeutic interventions.^16^, ^20^, ^23^, ^24^ The findings of the present study indicated only a minor role of ketone bodies as a source of energy in HF, suggesting that their beneficial effects in HF may be mediated by alternative mechanisms.^25^

When considering myocardial recovery, previous research has provided a link between overall cardiac bioenergetics and reverse remodelling in HF.^26^, ^27^ Notably, metrics of FA/Glc utilisation were associated with the ventricular response after LVAD implantation. As such, patients with more normal substrate preference (higher FA/Glc utilisation) showed a greater extent of reverse structural and functional adaption upon mechanical unloading. This finding is of particular interest as it implies a critical role of cardiac substrate use for the heart's ability to recover. Watson *et al*.^21^ previously demonstrated improvements in myocardial energetics and LV systolic function after intravenous lipid infusion in patients with non‐ischaemic HF. Furthermore, a recent study suggested that higher stroke work and reverse remodelling upon cardiac resynchronisation therapy may be mediated by normalisation of myocardial FA uptake.^22^ The authors hypothesised that the higher ATP efficiency (yield of ATP per 2‐carbon moiety of the used substrate) of FA oxidation compared to Glc use may have contributed to these beneficial effects, whereas the substrates' oxygen efficiency might be less relevant.^22^, ^28^ Indeed, the majority of LVAD patients in our study had non‐ischaemic cardiomyopathy where oxygen delivery is not considered a limiting factor. Therefore, the increased ATP availability associated with higher FA use might also explain the favourable adaptive response upon mechanical unloading observed in the present work. Collectively, the existing body of evidence suggests that manipulation of cardiac fuel use towards higher FA oxidation might represent a therapeutic strategy, and further studies evaluating this approach are warranted.

A major strength of the study is that computational models can be used to create personalised predictions based on patient‐specific information. Through this, high‐precision network simulations were performed to evaluate potential therapeutic targets for manipulation of myocardial substrate use. Interestingly, it was found that even modest increases in circulating FA levels could result in normalisation of FA/Glc ratios in HF. This might inform dose selection in future HF trials given the excessive elevation of FA levels reported after lipid infusion and corresponding risk of lipotoxicity.^21^

In addition, simulations indicated a potential role for carnitine supplementation to enhance FA utilisation in patients with reduced mitochondrial carnitine content. Indeed, carnitine concentration is reduced in the failing myocardium,^29^ and markers of impaired carnitine metabolism have been linked to adverse clinical outcomes.^30^ The results of this *in silico* work highlight the importance of carnitine for myocardial fuel choice and suggest possible value of repleting myocardial carnitine content as a disease‐modifying intervention in HF.

There are many future directions for this work. As our methodical framework requires myocardial tissue analyses, its application is restricted to clinical scenarios involving invasive procedures, such as heart surgeries or endomyocardial biopsies. Therefore, non‐invasive diagnostic markers reflecting the metabolic alterations identified by our *in silico* work are imperative for the translational potential of these results to be fully realised. Indeed, positron emission tomography is an established method to examine myocardial FA and Glc metabolism,^31^ which warrants further investigation with regard to its assessment in patients undergoing LVAD implantation. In addition, recent studies demonstrated the ability of hyperpolarised magnetic resonance to provide molecular‐resolved insights into myocardial substrate metabolism,^32^ and development of corresponding metabolic probes appears as another promising approach for the translation of our findings. Previous studies have shown that LVAD implantation may lead to improvements in myocardial and systemic metabolism.^18^, ^19^, ^33^, ^34^ If there is a link between these beneficial metabolic effects and myocardial recovery upon LVAD support awaits further investigation, and assessment of myocardial metabolism by computational modelling could yield important mechanistic insights.

Overall, this study introduced a computational method that creates a patient‐specific map of the dynamic myocardial metabolic network in HF. This provides a platform for integration of additional multidimensional data like information on cardiac function, electrophysiology, or genetic background. Thus, this work significantly contributes to the implementation of the ‘heart of the digital twin’ by enabling access to myocardial metabolism, one of the most complex domains within cardiac physiology.

### Limitations

Although this work utilised two of the largest myocardial proteomic datasets reported in human HF, the overall modest sample size represents a main limitation of the present study. Due to the retrospective study design, data had to be extracted from medical records which had been inputted during routine clinical care, without a standardised study protocol, making the results hypothesis‐generating. Analyses were carried out in myocardial biopsies, which may not adequately reflect intra‐organ heterogeneity (i.e. sampling error). Our approach focused on proteomic adaptions at cellular level but did not account for structural alterations within the tissue environment (e.g. coronary artery disease, microvascular dysfunction/rarefaction, cardiomyocyte hypertrophy, myocardial fibrosis). By assuming identical levels of circulating nutrients, hormones, and ions, we were able to study alterations in myocardial metabolism under standardised conditions. However, concentrations may vary across different cardiac regions due to local structural alterations. Additionally, the lack of patient‐specific plasma profiles means the impact of individual nutrient, hormone, and ion concentrations on myocardial metabolism was not considered and needs to be investigated in future research. Besides nutrient availability, metabolic activity depends on energetic demands (e.g. contraction, ion handling, self‐maintenance), and alterations in any of these functions will affect cardiac metabolic activity. While personalised estimates were used to assess overall metabolic activity based on blood pressure and heart rate, a more detailed consideration of individual alterations in various cardiac functions might improve/modify the results.

## Conclusions

In conclusion, computational modelling is presented as a novel personalised approach to map the metabolic network in human HF. Impaired energy production capacity and a shift in substrate preference represent general hallmarks of the failing myocardium that can be quantitatively assessed at the individual patient level using this analytical framework. Computational assessment of myocardial metabolism in HF may improve understanding of disease heterogeneity, individual risk stratification, and guidance of personalised clinical decision‐making in the future.

### Funding

This study was supported by the German Centre for Cardiovascular Research (DZHK; 81Z0100212), the German Cardiac Society (DGK07/2021), and the NIHR Oxford Biomedical Research Centre. The collaboration was supported by the Oxford Berlin Research Partnership, funded under the Excellence Strategy of the Federal Government and the Länder, by the Berlin University Alliance, and by the University of Oxford. Niklas Beyhoff is participant in the BIH Charité Clinician Scientist Program funded by the Charité ‐ Universitätsmedizin Berlin and the Berlin Institute of Health. István Baczkó was supported by the Hungarian National Research, Development and Innovation Office (TKP2021_EGA‐32 and K‐147212). Ulrich Kintscher is supported by the DZHK (BER 5.4 PR) and the Deutsche Forschungsgemeinschaft (DFG, German Research Foundation) – DFG‐KI 712/10‐1; SFB‐1470‐A09. Philipp Mertins and Nikolaus Berndt were funded by the Deutsche Forschungsgemeinschaft – SFB‐1470‐B05 (P.M.); SFB1470‐A08 (N.B.).

**Conflict of interest**: The patent application EP21174633 with the title ‘Computer assisted method for the evaluation of cardiac metabolism’ was filed by Charité ‐ Universitätsmedizin Berlin as the employer of Nikolaus Berndt and Titus Kühne who hold inventorship for this patent application. Nikolaus Berndt is founder and shareholder of Doppelganger Biosystem. All other authors have nothing to disclose.

## Supporting information


**Appendix S1.** Supporting Information.
