# Supplementary material for: Computational modelling of myocardial metabolism in patients with advanced heart failure
Source: Eur J Heart Fail. 2025 Jul 15;27(12):3411–22. doi: 10.1002/ejhf.3746 (PMC12803576; doi:10.1002/ejhf.3746)
Supplement: Supplementary file 1 — Appendix S1. Supporting Information. [file EJHF-27-3411-s001.pdf]

SUPPLEMENTAL MATERIAL

Supplemental Methods

Quantitative proteomics of myocardial tissue

Sample preparation

Myocardial samples were lysed in 200 µl lysis buffer containing 2% sodium dodecyl sulfate, 50 mM ammonium bicarbonate buffer, and EDTA-free cOmplete Protease Inhibitor Cocktail (Roche, Switzerland). Samples were homogenised at room temperature using a FastPrep-24 5G Bead Beating Grinder and Lysis System (MP Biomedicals, United States) with 10 cycles of 20 and 5 second pauses between cycles. After heating the samples for 5 minutes at 95°C, 5 freeze-thaw cycles were applied. 25 U of Benzonase (Merck, Germany) was added to each sample and after an incubation for 30 min the lysates were clarified by centrifugation at 16,000 g for 40 min at 4°C. Protein concentration was measured (DC Protein assay, Bio-Rad, United States) and 50 µg of each sample was further processed using the SP3 clean-up and digestion protocol as previously described.[1] Each sample was reduced with dithiothreitol (10 mM final; Sigma-Aldrich, United States) for 30 minutes, followed by alkylation with chloroacetamide (40 mM final, Sigma-Aldrich) for 45 minutes and quenching with dithiothreitol (20 mM final, Sigma-Aldrich). Beads (1 mg) and acetonitrile (70% final concentration) were added to each sample. After 20 minutes of incubation on an over-head rotor, the bead-bound protein was washed with 70% ethanol and 100% acetonitrile. 2µg sequence-grade Trypsin (Promega, United States) and 2 µg Lysyl Endopeptidase Lys-C (FUJIFILM Wako Pure Chemical Corporation, Japan) in 50 mM HEPES (pH 8) were added. After an fasting incubation at 37°C the peptides were collected, acidified with trifluoroacetic acid, and cleaned using the StageTips protocol.[2]

Liquid chromatography-mass spectrometry (LC-MS/MS) analyses

Peptide samples were eluted from StageTips (80% acetonitrile, 0.1% formic acid). After evaporating organic solvent, peptides were resolved in sample buffer (3% acetonitrile/ 0.1% formic acid). Peptide separation was performed on a 20-cm reversed-phase column (75 µm inner diameter, packed with ReproSil-Pur C18-AQ; 1.9 µm; Dr. Maisch GmbH, Germany) using a 200 min gradient with a 250 nl/min flow rate of increasing Buffer B concentration (from 2% to 60%) on a high-performance liquid chromatography system (ThermoScientific, United States). Peptides were measured on an Q Exactive HF-X Orbitrap instrument (ThermoScientific). Full scans were performed at 60K resolution using $3x10^6$ ion count target and a maximum injection time of 10 ms. MS^2 scans were acquired in Top 20 mode at 15K resolution with $1x10^5$ ion count target, 1.6 m/z isolation window and maximum injection time of 22 ms. Each sample was measured twice, and these two technical replicates were combined in subsequent data analyses.

Raw data processing

Data were analysed using MaxQuant software package (v1.6.3.4). The internal Andromeda search engine was used to search MS2 spectra against a decoy human UniProt database (HUMAN.2020-06) containing forward and reverse sequences. The search included variable modifications of oxidation (M), N-terminal acetylation, deamidation (N and Q) and fixed modification of carbamidomethyl cysteine. Minimal peptide length was set to six amino acids and a maximum of three missed cleavages was allowed. The false discovery rate was set to 1% for peptide and protein identifications. Unique and razor peptides were considered for quantification. Retention times were recalibrated based on the built-in nonlinear time-rescaling algorithm. A human heart library[1] was added to the raw file processing and MS2 identifications were transferred between runs with the “Match between runs” option, in which the maximal

retention time window was set to 0.7 min. The integrated LFQ [label-free quantitation] algorithm was applied. Resulting protein groups text files were filtered (exclusion of reverse hits, proteins only identified by site and contaminants, minimum valid value of 70%), intensity values were log2 transformed and missing values were imputed with low-intensity values simulating the detection limit of the mass spectrometer. Only samples containing <5% haemoglobin and <5% collagen fraction were included to control for potential blood contamination and presence of excessive scar tissue, respectively.

The mass spectrometry proteomics data have been deposited to the ProteomeXchange Consortium via the PRIDE partner repository with the dataset identifier PXD053721 (https://www.ebi.ac.uk/pride/archive/projects/PXD053721).

Computational modelling framework

Model overview

Development, parametrisation, validation, and first applications of the model were previously described.[3] A kinetic model of myocardial metabolism [CARDIOKIN1] was designed capturing a total of 296 metabolic processes (biochemical reactions) involved in the catabolism of FA, Glc, lactate/pyruvate, ketone bodies, and branched-chain amino acids, as well as the maintenance of endogenous energy stores (glycogen and triacylglycerol).[3] The model also includes the transmembrane transport of various ions, key electrophysiologic processes at the inner mitochondrial membrane, and the short-term regulatory effects of insulin and catecholamines on metabolic enzymes and transporters.[3] The temporal dynamics of the model variables (i.e., metabolite and ion concentrations) were described by first-order differential equations. The time variations of small ions were modelled based on kinetic equations of the Goldman-Hodgkin-Katz type.[3] Rate laws for enzymes and membrane transporters were taken from existing literature or derived from published experimental data.[3] Considered regulatory

mechanisms included the regulation of enzymes and transporters by reaction substrates and products, allosteric effectors, and reversible phosphorylation. For more detailed information on the applied kinetic equations and model parameters as well as their derivation, have been previously published.[3]

All computations were performed using MATLAB (release R2023b; MathWorks, United States).

Model parametrisation

Using this computational framework, personalised models of myocardial metabolism were built for each patient by incorporating the protein intensity profile of each individual's LV biopsy.[3,4] While the kinetic parameters of the rate laws are properties of the corresponding enzyme, the maximal enzyme/transporter activity $V_{\max}$ may vary across individuals depending on the enzyme abundance: $V_{\max} = k_{cat} \times E$ (with k_{cat} being the turnover rate of a single enzyme and E the enzyme concentration).[3] Based on this simple relationship, personalised models of myocardial metabolism were constructed for each patient by scaling the maximal activity of enzymes and transporters according to their individual proteomics-derived abundance in the subject's LV biopsy.[3,4]

$$V_{\max}^{subject} = V_{\max}^{normal} \frac{E^{subject}}{E^{control}}$$

with $E^{control}$ being the average protein intensity of the enzyme in the nonfailing control group and $E^{subject}$ being the protein intensity of the enzyme in the individual subject (nonfailing controls and HF group). For missing protein intensities, $V_{\max}$ was estimated by imputing the mean intensity from all samples within the same group. If no value was available for any sample in a group, it was assumed to be unchanged compared to the mean of the control group. The maximal enzyme/transporter activities of the reference model for the average nonfailing

heart ($V^{normal}_{\max}$) were obtained by fitting the model to collective evidence from experimentally observed physiology.[3]

Computation of metabolic capacities

Personalised models of myocardial metabolism were used to estimate individual capacities for the utilisation of the main substrates FA, Glc, lactate/pyruvate, ketone bodies, and branched-chain amino acids. ATP production, oxygen consumption, and uptake rates of energy-delivering substrates at different levels of myocardial workload were determined at different physiological and pathophysiological plasma metabolite concentrations as described previously.[3] Resting ATP production was computed via calculation of the individual's myocardial oxygen consumption (MV_{O2}), which can be estimated by following two-factor approximation[3,5]:

$$MV_{O2} = \text{heart rate} \times \text{systolic blood pressure} \times \text{proportionality factor } \gamma$$

If data on heart rate and/or blood pressure was not available, simulation of resting ATP production was performed considering normal values (heart rate 70/min, systolic blood pressure 135 mmHg).[3]

Metabolic control theory

The metabolic network's functionality depends on enzyme abundance, nutrient availability, and necessary cofactors. Since each component influences metabolic performance, we assessed individual effects on the FA/Glc ratio at rest.

Effect of enzyme abundance: To determine the impact of individual metabolic processes, their activity/the abundance of underlying enzymes was systematically altered by 10% for every patient, constructing distinct metabolic networks for each case. For each network, ATP

production capacity under fasting conditions was calculated as described previously.[3] Metabolic fluxes were then analysed to determine the FA/Glc ratio.

Effect of substrate availability: Substrate availability was adjusted by modifying the physiological fasting plasma profile (**Table S2**). Circulating FA levels were increased incrementally up to 1 mM (0.1 mM steps). For each patient and plasma profile, ATP production capacity was determined as previously described,[3] and the FA/Glc ratio was calculated.

Effect of carnitine availability: Free mitochondrial carnitine was varied between 0 and 4 mM, and free cytosolic carnitine between 0 and 0.4 mM for each patient. ATP production capacity under fasting conditions was recalculated for each variation,[3] and the resulting metabolic fluxes were used to compute the FA/Glc ratio at rest.

Confirmation cohort

Results obtained from the study cohort were compared with an external confirmation cohort from Australia. The confirmation cohort included myocardial proteomics of patients with advanced HF (nearly half of which had ischaemic cardiomyopathy) together with age- and sex-matched nonfailing controls.[6] Detailed information on patient characteristics, tissue collection, sample processing, and proteomic analyses were reported previously.[6] The confirmation dataset used in the present study comprised myocardial proteomics from 44 advanced HF samples and 28 nonfailing samples (sample selection and group assignment based on documents provided by the authors of the original study[6]).

Data-independent acquisition mass spectrometry RAW data was retrieved from the repository and reanalysed with Spectronaut (version 16; Biognosys, Switzerland) using a library-based approach, comparable to the original study.[6] The Q-sparse filter (for protein identification) and a global normalisation strategy (Quant 2.0) were applied. Proteins annotated as 'single hits' were filtered out before statistical analyses. Finally, intensity profiles of relevant proteins were

incorporated into the modelling framework to build customized models of myocardial metabolism for the confirmation cohort as outlined in the corresponding methods section.

Compared to the study cohort, protein coverage was slightly lower in the confirmation dataset (2,812±37 proteins quantified per sample; **Figure S1B**). Expression patterns of the 304 proteins used for computational model generation are shown in **Figure S2B**.

Supplemental Figures

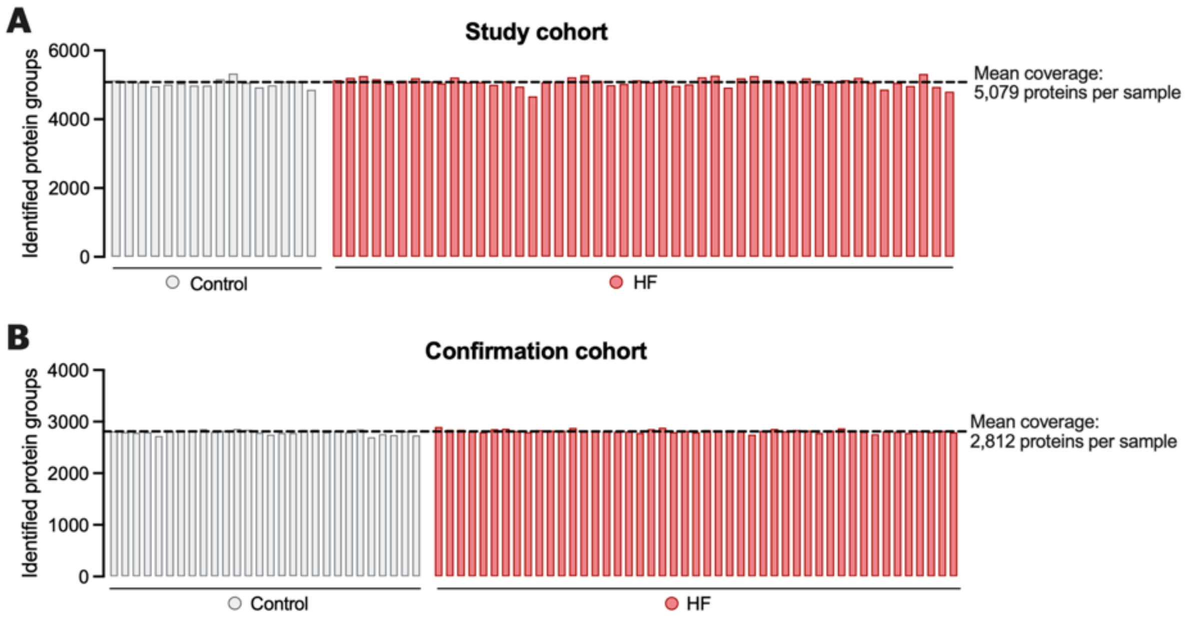

Figure S1: Uniform proteome coverage across studies. Number of proteins quantified per individual sample in the (**A**) study cohort and (**B**) confirmation cohort. Only proteins were counted that were quantified in >70% of samples in each cohort.

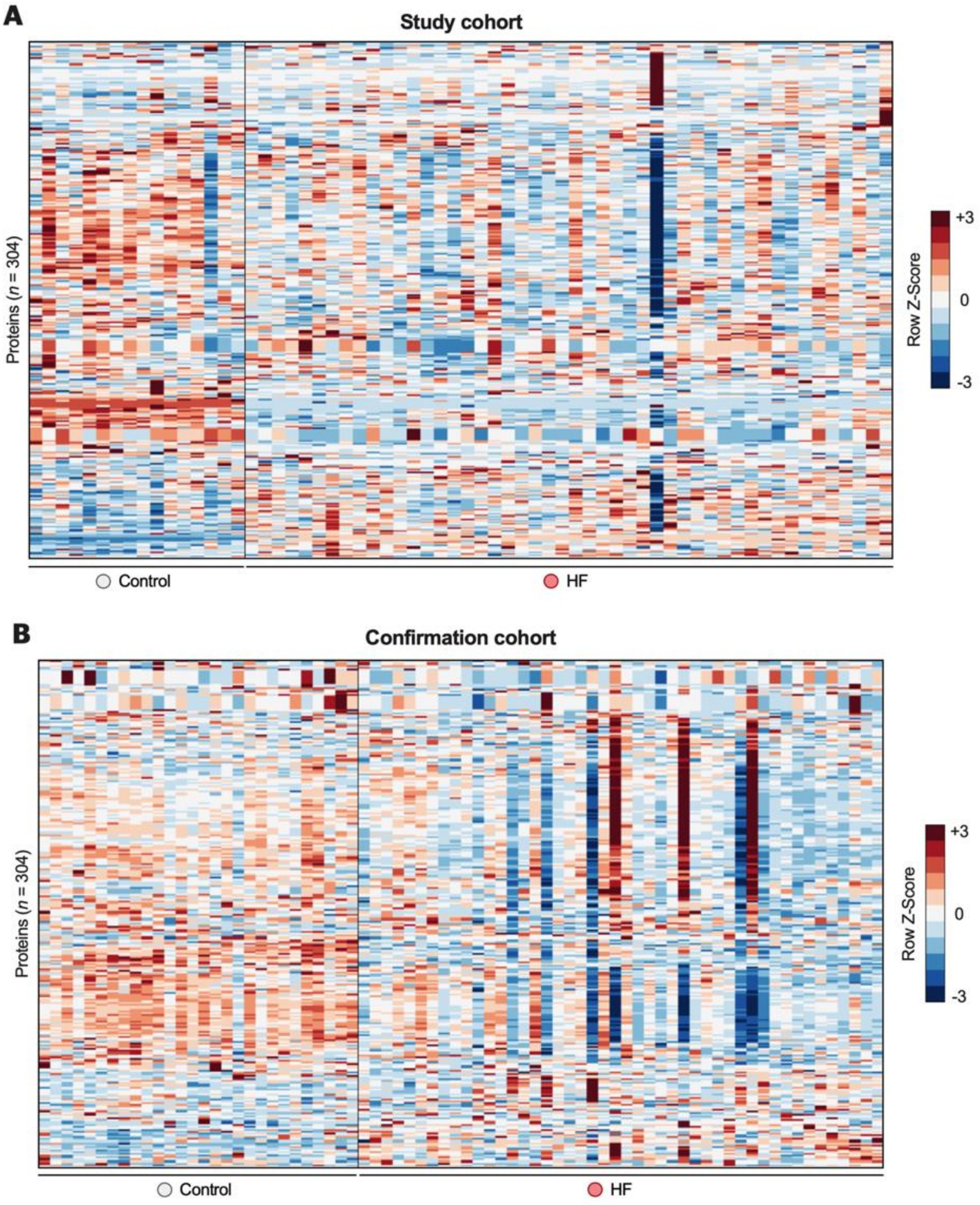

Figure S2: Individual expression patterns of the 304 proteins involved in myocardial metabolism used for computational model generation. Heatmap illustrating standardised protein expression at single patient-level in (**A**) the study cohort and (**B**) the confirmation cohort.

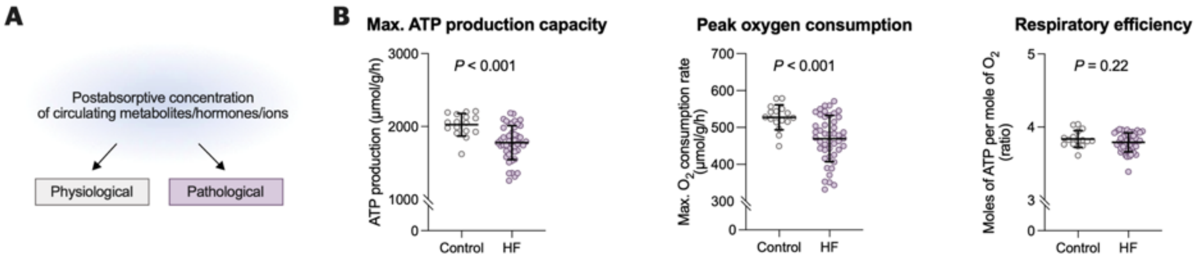

Figure S3: Myocardial metabolic capacities considering differences in fasting plasma concentrations. **A**, Outline of simulation experiment. Computations were performed considering reported differences in circulating metabolite/hormone/ion concentrations at fasting state between healthy controls (physiological concentrations) and patients with heart failure (pathological concentrations)[7]. **B**, Maximal ATP production capacity, peak oxygen consumption, and respiratory efficiency considering fasting substrate concentrations as reported in **A**.

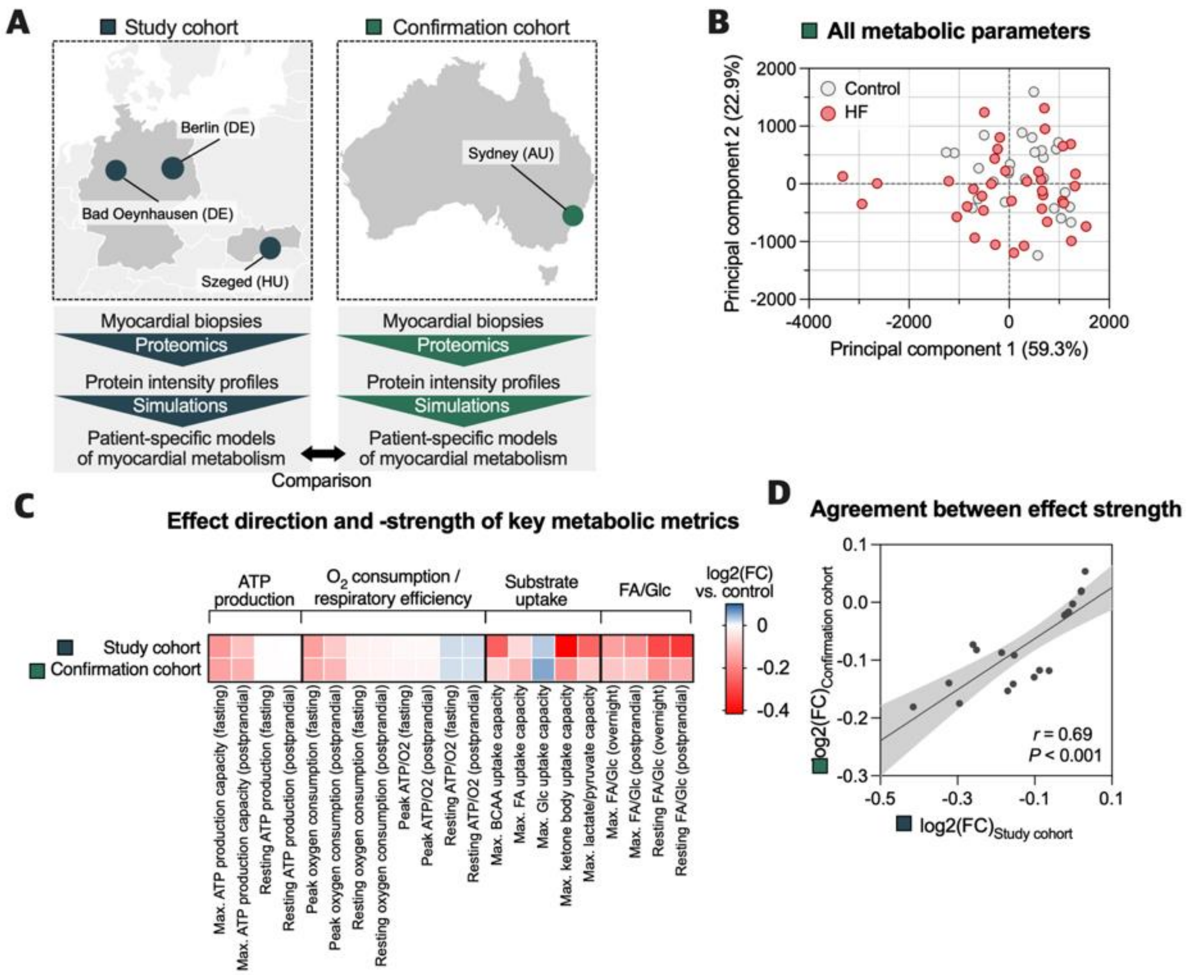

Figure S4: External confirmation of study results. A, Findings derived from the study cohort (Europe) were validated in a confirmation cohort of patients with advanced HF together with corresponding controls (Australia). **B**, Principal component analysis of key metabolic readouts ($n = 118$) in the confirmation cohort. **C**, Heat map showing the difference in various key metabolic metrics between HF and control subjects in the two cohorts. **D**, Correlation analysis of the relative difference in various key metabolic metrics between HF and controls in the study cohort and the confirmation cohort, respectively (Pearson's correlation coefficient *r*).

Supplemental Tables

#	Metabolic parameter
1	maximal atp production (fasting)
2	maximal o2 consumption (fasting)
3	max.atp/o2 ratio (fasting)
4	atp production rest (fasting)
5	oxygen consumption rest (fasting)
6	atp/o2 ratio rest (fasting)
7	atp production middle load (fasting)
8	oxygen consumption middle load (fasting)
9	atp/o2 ratio middle load (fasting)
10	atp production high load (fasting)
11	oxygen consumption high load (fasting)
12	atp/o2 ratio high load (fasting)
13	atp production max load (fasting)
14	oxygen consumption max load (fasting)
15	atp/o2 ratio max load (fasting)
16	glucose rest (fasting)
17	ketone bodies rest (fasting)
18	lactate/pyruvate rest (fasting)
19	fatty acids rest (fasting)
20	bcaa rest (fasting)
21	glucose middle load (fasting)
22	ketone bodies mid middle (fasting) load
23	lactate/pyruvate middle load (fasting)
24	fatty acids middle load (fasting)
25	bcaa middle load (fasting)
26	glucose high load (fasting)
27	ketone bodies high load (fasting)
28	lactate/pyruvate high load (fasting)
29	fatty acids high load (fasting)
30	bcaa high load (fasting)
31	glucose max load (fasting)
32	ketone bodies max load (fasting)
33	lactate/pyruvate max load (fasting)
34	fatty acids max load (fasting)
35	bcaa max load (fasting)
36	fatty acid/glucose ratio rest (fasting)
37	fatty acid/glucose ratio middle load (fasting)
38	fatty acid/glucose ratio high load (fasting)
39	fatty acid/glucose ratio max load (fasting)
40	rel. contribution glucose rest (fasting)
41	rel. contribution ketones rest (fasting)
42	rel. contribution lactate rest (fasting)
43	rel. contribution fatty acids rest (fasting)
44	rel. contribution bcaa rest (fasting)
45	rel. contribution glucose middle load (fasting)
46	rel. contribution ketones middle load (fasting)
47	rel. contribution lactate middle load (fasting)
48	rel. contribution fatty acids middle load (fasting)
49	rel. contribution bcaa high load (fasting)
50	rel. contribution glucose high load (fasting)
51	rel. contribution ketones high load (fasting)
52	rel. contribution lactate high load (fasting)

53	rel. contribution fatty acids high load (fasting)
54	rel. contribution bcaa high load (fasting)
55	rel. contribution glucose max load (fasting)
56	rel. contribution ketones max load (fasting)
57	rel. contribution lactate max load (fasting)
58	rel. contribution fatty acids max load (fasting)
59	rel. contribution bcaa max load (fasting)
60	maximal atp production postprandial
61	maximal o2 consumption postprandial
62	atp/o2 ratio postprandial
63	atp production rest (postprandial)
64	oxygen consumption rest (postprandial)
65	atp/o2 ratio rest (postprandial)
66	atp production middle load (postprandial)
67	oxygen consumption middle load (postprandial)
68	atp/o2 ratio middle load (postprandial)
69	atp production high load (postprandial)
70	oxygen consumption high load (postprandial)
71	atp/o2 ratio high load (postprandial)
72	atp production max load (postprandial)
73	oxygen consumption max load (postprandial)
74	atp/o2 ratio max load (postprandial)
75	glucose rest (postprandial)
76	ketone bodies rest (postprandial)
77	lactate/pyruvate rest (postprandial)
78	fatty acids rest (postprandial)
79	bcaa rest (postprandial)
80	glucose middle load (postprandial)
81	ketone bodies middle load (postprandial)
82	lactate/pyruvate middle load (postprandial)
83	fatty acids middle load (postprandial)
84	bcaa middle load (postprandial)
85	glucose high load (postprandial)
86	ketone bodies high load (postprandial)
87	lactate/pyruvate high load (postprandial)
88	fatty acids high load (postprandial)
89	bcaa high load (postprandial)
90	glucose max load (postprandial)
91	ketone bodies max load (postprandial)
92	lactate/pyruvate max load (postprandial)
93	fatty acids max load (postprandial)
94	bcaa max load (postprandial)
95	fatty acid/glucose ratio rest (postprandial)
96	fatty acid/glucose ratio middle load (postprandial)
97	fatty acid/glucose ratio high load (postprandial)
98	fatty acid/glucose ratio max load (postprandial)
99	rel. contribution glucose rest (postprandial)
100	rel. contribution ketones rest (postprandial)
101	rel. contribution lactate rest (postprandial)
102	rel. contribution fatty acids rest (postprandial)
103	rel. contribution bcaa rest (postprandial)
104	rel. contribution glucose middle load (postprandial)
105	rel. contribution ketones middle load (postprandial)
106	rel. contribution lactate middle load (postprandial)
107	rel. contribution fatty acids middle load (postprandial)

108	rel. contribution bcaa high load (postprandial)
109	rel. contribution glucose high load (postprandial)
110	rel. contribution ketones high load (postprandial)
111	rel. contribution lactate high load (postprandial)
112	rel. contribution fatty acids high load (postprandial)
113	rel. contribution bcaa high load (postprandial)
114	rel. contribution glucose max load (postprandial)
115	rel. contribution ketones max load (postprandial)
116	rel. contribution lactate max load (postprandial)
117	rel. contribution fatty acids max load (postprandial)
118	rel. contribution bcaa max load (postprandial)

Table S1: List of the 118 key metabolic parameters used for principal component analyses. Corresponding metabolic functions, metabolic processes, and abbreviations are detailed elsewhere[3].

	**Physiologic postabsorptive (fasting) state[3]**	**Postabsorptive (fasting) state in HF[7]**	**Physiologic postprandial state[3]**	**Diabetic state[8–11]**
**Glc**	5.8	5.2	7.8	9.7
**FA**	0.5	0.603	0.2	0.6
**Lactate**	0.8	0.74	2	0.8
**Glutamine**	0.5	0.4	0.5	0.5
**Valine**	0.2	0.6	0.4	0.4
**Leucine**	0.15	0.2	0.4	0.4
**Isoleucine**	0.06	0.15	0.2	0.2
**β-hydroxybutyrate**	0.08	0.2003	0	0.131
**Acetoacetate**	0.04	0.0668	0	0.032

Table S2: Concentration of metabolites, hormones, and ions used for computations and simulations. Concentrations in mM.

Supplemental References

1. Nordmeyer S, Kraus M, Ziehm M, Kirchner M, Schafstedde M, Kelm M, Niquet S, Stephen MM, Baczko I, Knosalla C, Schapranow M-P, Dittmar G, Gotthardt M, Falcke M, Regitz-Zagrosek V, Kuehne T, Mertins P. Disease- and sex-specific differences in patients with heart valve disease: a proteome study. *Life Sci Alliance* 2023;**6**:e202201411.

2. Rappsilber J, Ishihama Y, Mann M. Stop and go extraction tips for matrix-assisted laser desorption/ionization, nanoelectrospray, and LC/MS sample pretreatment in proteomics. *Anal Chem* 2003;**75**:663–670.

3. Berndt N, Eckstein J, Wallach I, Nordmeyer S, Kelm M, Kirchner M, Goubergrits L, Schafstedde M, Hennemuth A, Kraus M, Grune T, Mertins P, Kuehne T, Holzhütter H-G. CARDIOKIN1: Computational Assessment of Myocardial Metabolic Capability in Healthy Controls and Patients With Valve Diseases. *Circulation* 2021;**144**:1926–1939.

4. Berndt N, Bulik S, Wallach I, Wünsch T, König M, Stockmann M, Meierhofer D, Holzhütter H-G. HEPATOKIN1 is a biochemistry-based model of liver metabolism for applications in medicine and pharmacology. *Nat Commun* 2018;**9**:2386.

5. Nelson RR, Gobel FL, Jorgensen CR, Wang K, Wang Y, Taylor HL. Hemodynamic Predictors of Myocardial Oxygen Consumption During Static and Dynamic Exercise. *Circulation* 1974;**50**:1179–1189.

6. Li M, Parker BL, Pearson E, Hunter B, Cao J, Koay YC, Guneratne O, James DE, Yang J, Lal S, O'Sullivan JF. Core functional nodes and sex-specific pathways in human ischaemic and dilated cardiomyopathy. *Nat Commun* 2020;**11**:2843.

7. Lommi J, Kupari M, Koskinen P, Näveri H, Leinonen H, Pulkki K, Härkönen M. Blood ketone bodies in congestive heart failure. *J Am Coll Cardiol* 1996;**28**:665–672.

8. Garcia E, Shalaurova I, Matyus SP, Oskardmay DN, Otvos JD, Dullaart RPF, Connelly MA. Ketone Bodies Are Mildly Elevated in Subjects with Type 2 Diabetes Mellitus and Are Inversely Associated with Insulin Resistance as Measured by the Lipoprotein Insulin Resistance Index. *J Clin Med* 2020;**9**:321.

9. Crawford SO, Hoogeveen RC, Brancati FL, Astor BC, Ballantyne CM, Schmidt MI, Young JH. Association of blood lactate with type 2 diabetes: the Atherosclerosis Risk in Communities Carotid MRI Study. *Int J Epidemiol* 2010;**39**:1647–1655.

10. Hall SEH, Saunders J, S□nksen PH. Glucose and free fatty acid turnover in normal subjects and in diabetic patients before and after insulin treatment. *Diabetologia* 1979;**16**:297–306.

11. Meikle PJ, Wong G, Barlow CK, Weir JM, Greeve MA, MacIntosh GL, Almasy L, Comuzzie AG, Mahaney MC, Kowalczyk A, Haviv I, Grantham N, Magliano DJ, Jowett JBM, Zimmet P, Curran JE, Blangero J, Shaw J. Plasma Lipid Profiling Shows Similar Associations with Prediabetes and Type 2 Diabetes. Maya-Monteiro CM, ed. *PLoS ONE* 2013;**8**:e74341.
